# Functional requirements for inhibitory signal transmission by the immunomodulatory receptor CD300a

**DOI:** 10.1186/1471-2172-13-23

**Published:** 2012-04-26

**Authors:** Karen E DeBell, Venkateswara R Simhadri, John L Mariano, Francisco Borrego

**Affiliations:** 1 Laboratory of Molecular and Developmental Immunology, Division of Monoclonal Antibodies HFD-123, OBP/CDER/FDA, 29 Lincoln Drive; Bldg 29B, Room 3NN18, Bethesda, MD 20892, USA

## Abstract

**Background:**

Activation signals can be negatively regulated by cell surface receptors bearing immunoreceptor tyrosine-based inhibitory motifs (ITIMs). CD300a, an ITIM bearing type I transmembrane protein, is expressed on many hematopoietic cells, including subsets of lymphocytes.

**Results:**

We have taken two approaches to further define the mechanism by which CD300a acts as an inhibitor of immune cell receptor signaling. First, we have expressed in Jurkat T cells a chimeric receptor consisting of the extracellular domains of killer-cell immunoglobulin-like receptor (KIR)2DL2 fused to the transmembrane and cytoplasmic segments of CD300a (KIR-CD300a) to explore surrogate ligand-stimulated inhibition of superantigen stimulated T cell receptor (TCR) mediated cell signaling. We found that intact CD300a ITIMs were essential for inhibition and that the tyrosine phosphorylation of these ITIMs required the src tyrosine kinase Lck. Tyrosine phosphorylation of the CD300a ITIMs created docking sites for both src homology 2 domain containing protein tyrosine phosphatase (SHP)-1 and SHP-2. Suppression of SHP-1 and SHP-2 expression in KIR-CD300a Jurkat T cells with siRNA and the use of DT40 chicken B cell lines expressing CD300a and deficient in several phosphatases revealed that SHP-1, but not SHP-2 or the src homology 2 domain containing inositol 5’ phosphatase SHIP, was utilized by CD300a for its inhibitory activity.

**Conclusion:**

These studies provide new insights into the function of CD300a in tuning T and B cell responses.

## Background

An appropriate immune response requires a fine balance between a multitude of activating and inhibitory signals and the loss of the ability to limit positive signaling can result in autoreactivity and excessive inflammation [1,2]. A diverse array of inhibitory receptors participates in the negative control of the immune response. A characteristic of many of these receptors is a consensus amino acid sequence in their cytoplasmic tail, i.e. the immunoreceptor tyrosine-based inhibitory motif (ITIM) [3-8]. Ligand interaction with these receptors results in ITIM tyrosine phosphorylation, usually by a src family kinase, providing sites for binding proteins *via* their src-homology 2 (SH2) domains [9-14]. Proteins containing consensus sequences for interaction with phosphorylated ITIMs include the SH2 domain-containing tyrosine phosphatase (SHP)-1, SHP-2, and the SH2 domain-containing inositol 5’-phosphatase (SHIP) [10,13-16]. The recruitment of phosphatases to the phosphorylated ITIMs results in their activation and the subsequent dephosphorylation of their substrates, leading to the down-regulation of activation signals [9-14]. Although several targets of these phosphatases have been proposed, the specific pathways and mechanisms by which each phosphatase participates in the signaling cascade downstream from the inhibitory receptors remain incompletely understood [17-19].

CD300a is one of the seven members of the CD300 family of leukocyte surface receptors that are encoded by genes clustered in human chromosome 17q25 [20]. Like the other members of the CD300 family, CD300a is a type I transmembrane protein, with a single IgV-like extracellular region and three classical and one non-classical ITIMs in its cytoplasmic tail [20]. The CD300a gene has undergone a very significant positive selection, suggesting an essential requirement for the host to maintain its function throughout evolution [21,22]. CD300a is expressed on cells of both the myeloid and lymphoid lineages [20]. The clinical relevance of this receptor is demonstrated in reports showing the association of a non-synonymous polymorphism within the Ig-V domain with the development of psoriasis [23], the implication in the development of Alzheimer’s disease by genome wide association studies [24], the down-regulation of CD300a expression on B cells from HIV-1 infected patients [25], and the proposed use of CD300a as a biomarker that can differentiate ulcerative colitis from Crohn’s disease and non-inflammatory diarrhea [26] and for the detection of minimal residual disease in acute lymphoblastic leukemia [27].

*In vitro* studies have shown that CD300a ligation can inhibit NK cell mediated cytotoxicity [28,29], FcϵRI mediated activation of mast cells [30], FcγRIIa mediated reactive oxygen species production and Ca^2+^ flux in neutrophils [31] and eosinophils responses to eotaxin, GM-CSF and IL-5 [32]. Additionally, it has been shown to inhibit both B cell receptor (BCR) and T cell receptor (TCR) mediated Ca^2+^ mobilization and NFAT mediated transcriptional activity [25,33]. Furthermore, *in vivo* studies in mice have shown that CD300a is able to reverse remodeling and airway inflammation in a model of experimental asthma [34], to abrogate IgE mediated allergic reactions [35] and to inhibit stem cell factor (SCF) induced anaphylaxis [36]. Various mechanisms of the CD300a mediated inhibitory signaling have been proposed. Several publications have shown that phosphorylated CD300a is able to recruit different phosphatases depending on the examined cell type, although genetic evidences for the direct involvement of any phosphatase in the delivery of CD300a mediated inhibitory signal is lacking. For example, treatment of human NK cells with pervanadate led to tyrosine phosphorylation of CD300a and its association with both SHP-1 and SHP-2 [28], while in eosinophils cross-linking of the receptor with monoclonal antibodies (mAb) recruited SHP-1 but not SHP-2 [32]. In mast cells, after pervanadate treatment, SHP-1 and SHIP, but not SHP-2 co-precipitated with CD300a, while upon mAb driven cross-linking, SHIP, but not SHP-1 associated with CD300a [30]. Also in mast cells, precipitation of CD300a from cells treated with an anti-Kit-CD300a bispecific antibody induced its tyrosine phosphorylation and the recruitment of SHIP, but not SHP-1 [36].

In T and B lymphocytes the expression of CD300a is restricted to certain subsets [25,33,37,38]. Although it has been previously shown that ligation of CD300a with mAb inhibits BCR [25] and TCR mediated signals [33], the basis for this inhibition is not known. In this study we investigate the structural and functional requirements for CD300a mediated inhibitory signaling in B and T cells. Importantly, we establish a physiologically relevant model in which we explore ligand driven functions of CD300a. To accomplish this, a KIR-CD300a chimera was expressed in Jurkat T cells. Mixing these cells with MHC class I matched antigen presenting cells that were loaded with superantigen allowed us to determine the importance of the CD300a ITIMs, the means by which they are phosphorylated and the phosphatases that subsequently associate with them. Further studies, using DT40 B cell lines and siRNA mediated knock down of SHP-1 and SHP-2 in Jurkat T cells, were performed to discriminate among signaling intermediates utilized by CD300a.

## Results

### Intact ITIM motifs are required for CD300a mediated inhibitory signal

**Figure 1:**
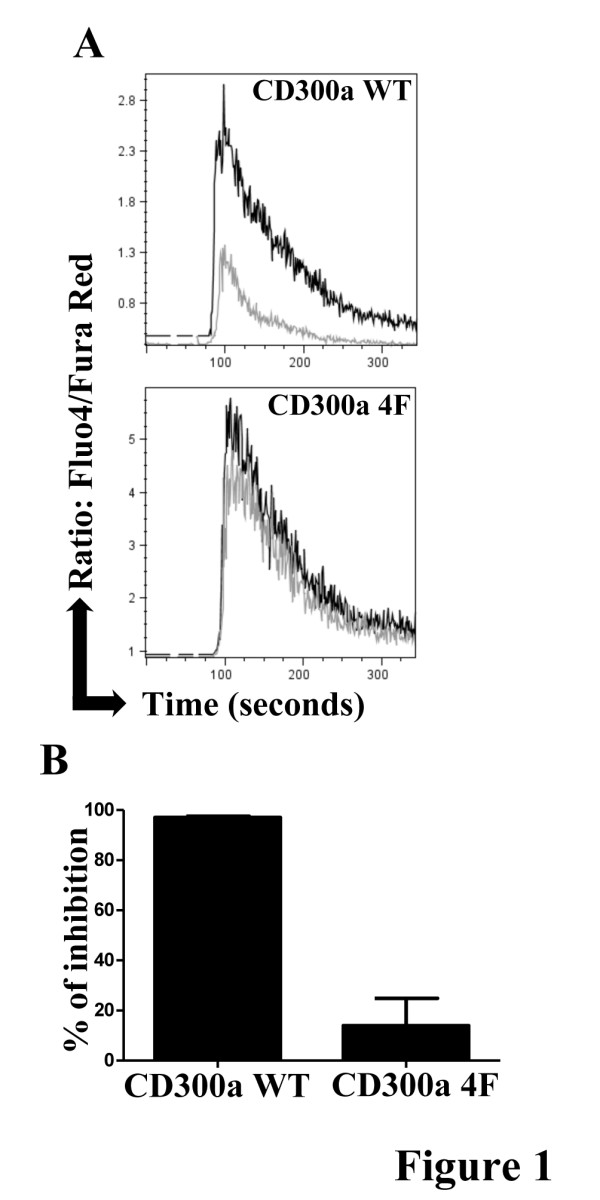
**The ITIMs of CD300a are essential for the inhibition of BCR stimulated activation.** (**A**) DT40 chicken B cells expressing CD300a WT or CD300a 4F were loaded with Fluo-4 and Fura-Red. Cells were stimulated with anti-chicken BCR plus isotype control antibody (black line) or anti-chicken BCR plus anti-CD300a mAb (grey line) for 30 seconds and then co-crosslinked with a secondary antibody (GAM). Fluorescence emission was measured in a flow cytometer. Ca^2+^ mobilization is expressed as the ratio of Fluo-4/Fura-Red as a function of time. These results are representative of three independent experiments. (**B**) DT40 chicken B cells expressing CD300a WT or CD300a 4F were transiently transfected with a NFAT luciferase reporter plasmid and stimulated with GAM plus anti-chicken BCR plus isotype control or anti-chicken BCR plus anti-CD300a mAb. The measured luciferase activity was normalized to the activity obtained with cells treated with PMA plus ionomycin. Data are presented as percentage of inhibition of CD300a vs. isotype control and they are the average ± SEM for three separate experiments.

Recently, we have demonstrated that the immunomodulatory receptor CD300a is expressed in certain subsets of human B and T cells and that it functions as a negative regulator of B and T cell signaling [25,33,38]. To explore the structural requirements for the CD300a mediated inhibitory signal, we have engineered plasmids encoding the CD300a receptor that have the tyrosine residues in the four ITIMs mutated to phenylalanine. The DT40 chicken B cell line was stably transfected with plasmids encoding the wild type CD300a receptor (CD300a WT) or the CD300a tyrosine to phenylalanine mutant receptor (CD300a 4F). We then examined the inhibitory effects of CD300a ligation on two BCR mediated events. As we have previously shown [25], coligation of the BCR with CD300a WT using mAb, resulted in a decreased rise of intracellular Ca^2+^ and a diminished NFAT transcriptional activity when compared with ligation of the BCR alone. However, when the experiments were performed with DT40 chicken B cells expressing CD300a 4F, no decrease in these BCR mediated events was observed (Figure 1). These results indicate that CD300a mediated inhibition of BCR driven signals is dependent on intact ITIMs.

### The intracellular tail of CD300a inhibits superantigen mediated activation of T cells

**Figure 2:**
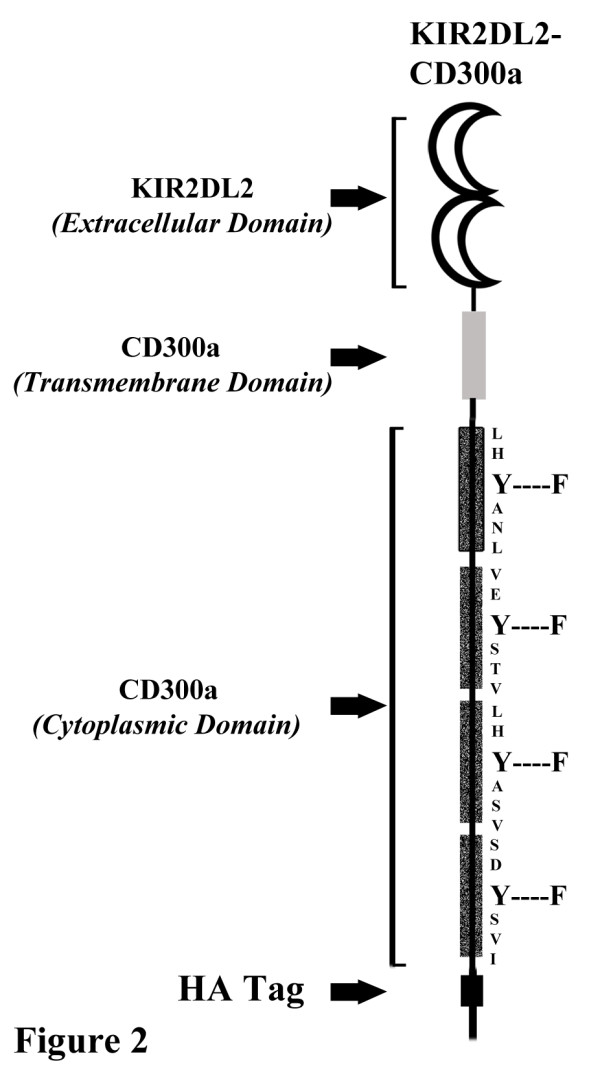
**Schematic representation of the chimeric KIR-CD300a receptors.** Structure of the chimeric molecules bearing the extracellular domain of KIR2DL2 and the transmembrane and cytoplasmic domains of CD300a is represented. The construct is fused to the hemagglutinin (HA) tag at the C-terminus. The shady dotted regions in the cytoplasmic tail correspond to the ITIMs in the CD300a receptor. The tyrosine in each of the 4 ITIMs was mutated to phenylalanine to generate the mutant chimeric construct KIR-CD300a 4F.

The above results and those published by others have shown that ligation of CD300a with mAb delivers an inhibitory signal in a variety of cell types [25,28-32,36]. We sought to investigate the inhibitory signaling potential of CD300a in a system that, instead, relies on receptor-ligand interaction. To do that we established stably transfected Jurkat T cell lines expressing a chimeric receptor that retains the transmembrane segment and the intracellular tail of CD300a but substitutes the extracellular portion of the receptor with that of KIR2DL2 whose ligands are the MHC Class I molecules HLA-Cw1, -Cw3, -Cw7 and -Cw8 [39]. In addition, an HA tag was added at the C terminal end. Two Jurkat T cell lines were established: KIR-CD300a WT, which conserves the wild type sequence of the intracellular tail of CD300a, and KIR-CD300a 4F, that has the tyrosine residues of the four CD300a ITIMs mutated to phenylalanine (Figure 2).

**Figure 3:**
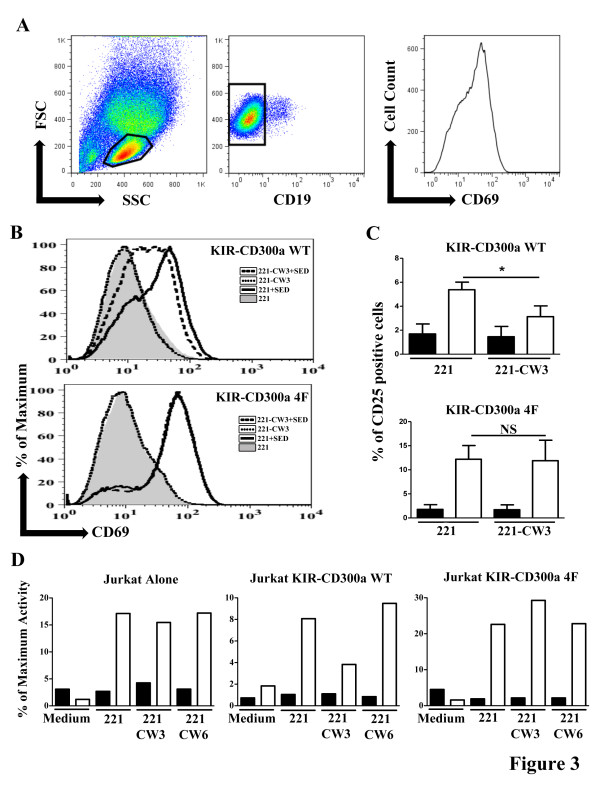
**KIR-CD300a mediates inhibition of SED stimulated Jurkat T cell activation.** (**A**) Gating strategy for assessing CD69 expression on superantigen activated cells. Jurkat T cells were electronically gated by size and forward scatter and by the absence of expression of CD19, a marker of 721.221 cells, and then the expression of CD69 was assessed. The gating strategy for assessing CD25 expression was the same. (**B**) KIR CD300a WT and KIR-CD300a 4F Jurkat T cells were co-cultured with 721.221 or 721.221-Cw3 cells, loaded or not with SED. Cultures were harvested and Jurkat T cells were assessed for CD69 expression by flow cytometry. Results are representative of three independent experiments. (**C**) Cells were cultured as in B, loaded (white bar) or not (black bars) with SED, and the expression of CD25 was assessed. The bar graph represents average ± SEM of the percentage of CD25+ Jurkat T cells. Results are from three independent experiments. (**D**) Untransfected E6.1, KIR-CD300a WT and KIR-CD300a 4F Jurkat T cells were transiently transfected with a NFAT luciferase reporter plasmid. Following coculture with 721.221, 721.221-Cw3 and 721.221-Cw6 loaded (withe bars) or not (black bars) with SED, cells were lysed and supernatants were assayed for luciferase activity. Data were normalized by the activity obtained with cells treated with PMA plus ionomycin. Results are representative of three independent experiments.

To study the ability of KIR-CD300a to inhibit TCR mediated signaling, we utilized a system that relies on the activation of Jurkat T cells by the bacterial superantigen SED, which binds the TCR Vβ chain. In our experimental design, SED is presented by MHC class II molecules expressed on the human B cell line 721.221. When Jurkat T cells were stimulated with the HLA-C negative 721.221 cells loaded with SED, an increase in the expression of the activation marker CD69 was observed. This occurred whether or not the Jurkat T cells expressed the KIR-CD300a WT or the KIR-CD300a 4F chimeric receptors. However, when SED was presented by 721.221 cells expressing the KIR2DL2 ligand HLA-Cw3 (721.221-Cw3), we observed a significant reduction in the upregulation of CD69 expression by KIR-CD300a WT Jurkat T cells. Conversely, presentation of SED by 721.221-Cw3 cells did not affect the upregulation of CD69 expression on KIR-CD300a 4F Jurkat T cells (Figure 3A and B). Similar results were obtained when we measured the expression of another activation marker, i.e. CD25 (Figure 3C). These results indicate that the intracellular tail of CD300a is responsible for inhibiting superantigen mediated activation signals, and confirm that the inhibitory signal requires intact ITIMs.

To further prove that the intracellular tail of CD300a is responsible for the inhibitory signal we performed additional experiments measuring NFAT transcriptional activity. We transiently transfected the E6.1 Jurkat T cell line and the KIR-CD300a WT and KIR-CD300a 4F expressing Jurkat T cells with a plasmid encoding the luciferase reporter gene under the control of a NFAT dependent promoter. Cells were stimulated through the TCR with SED presented by 721.221, 721.221-Cw3 and 721.221-Cw6 cells. The MHC class I molecule HLA-Cw6 is not a ligand for KIR2DL2 [39]. Results in Figure 3D showed that there was a decrease in the NFAT transcriptional activity only when KIR-CD300a WT Jurkat T cells were stimulated with SED solely presented by 721.221-Cw3 cells, and not 721.221 or 721.221-Cw6 cells. These results confirmed that the inhibition of superantigen mediated activation of Jurkat T cells required both the specific interaction between KIR2DL2 with its ligand, HLA-Cw3, and an intact CD300a intracellular tail.

### The src kinase lck is responsible for the tyrosine phosphorylation of CD300a on Jurkat T cells

**Figure 4:**
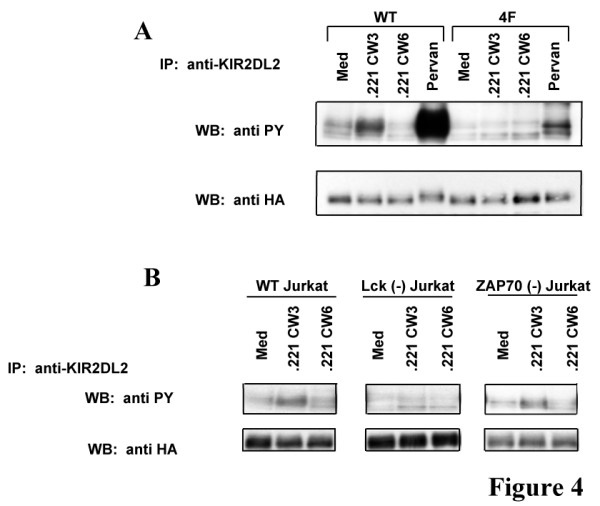
**Tyrosine phosphorylation of CD300a requires the src kinase Lck.** (**A**) KIR-CD300a WT and KIR-CD300a 4F Jurkat T cells were stimulated with medium or pervanadate for 3 minutes, or mixed with 721.221-Cw3 or 721.221-Cw6 and incubated at 37°C for 5 minutes. Cell lysates were immunopreciprecipitated with anti-KIR2DL2 (clone GL183) mAb and blotted separately for phosphotyrosine and HA. Results are representative of five independent experiments. (**B**) E6.1 Jurkat cells, Jurkat cells deficient in Lck or deficient in ZAP-70 were transiently transfected with a plasmid encoding KIR-CD300a and incubated at 37°C with 721.221-Cw3 and 721.221-Cw6 for 5 minutes. Cell lysates were immunoprecipitated with anti-KIR2DL2 (clone GL183) mAb and blotted separately for phosphotyrosine and HA. Results are representative of three independent experiments.

Interaction of ITIM containing receptors with their ligands leads to ITIM tyrosine phosphorylation. To demonstrate that CD300a ITIMs are tyrosine phosphorylated in response to KIR2DL2 ligand in our experimental system, KIR-CD300a WT and KIR-CD300a 4F Jurkat T cells were mixed with 721.221-Cw3 and 721.221-Cw6 cells and then anti-KIR2DL2 immunoprecipitates from cell lysates were examined by western blot analysis (Figure 4). We observed that KIR-CD300a WT was tyrosine phosphorylated when Jurkat T cells interacted with 721.221-Cw3 cells but not with 721.221-Cw6 cells. Pervanadate treatment was used as a positive control. As expected, co-culture of KIR-CD300a 4F Jurkat T cells with 721.221-Cw3 cells did not cause KIR-CD300a 4F phosphorylation (Figure 4A).

It has been previously described that the src kinase Lck is required for KIR tyrosine phosphorylation [9]. In our experimental system, in order to identify the kinase responsible for phosphorylation of CD300a ITIMs, the E6.1 Jurkat T cell line and the Jurkat T cell lines deficient in Lck or ZAP-70 were transiently transfected with a plasmid encoding KIR-CD300a WT. These cells were mixed with 721.221-Cw3 or 721.221-Cw6 cells and tyrosine phosphorylation was assessed in KIR2DL2 immunoprecipitates (Figure 4B). Co-incubation of 721.221-Cw3 cells with either the E6.1 Jurkat T cell line or the ZAP-70 deficient cells led to tyrosine phosphorylation of KIR-C300a, indicating that ZAP-70 is not necessary for tyrosine phosphorylation of the intracellular tail of CD300a. However, when Jurkat T cells deficient in Lck were incubated with 721.221-Cw3, tyrosine phosphorylation of KIR-CD300a was not observed in any of the immunoprecipitates. As expected, co-incubation of any Jurkat T cell lines with 721.221-Cw6 cells did not stimulate tyrosine phosphorylation of KIR-CD300a WT. Together, these results show that ligand-receptor interaction leads to tyrosine phosphorylation of the CD300a ITIMs in the absence of an activation signal, and that the src kinase Lck is responsible for tyrosine phosphorylation of the CD300a ITIM motifs in Jurkat T cells.

### Both SHP-1 and SHP-2 bind to CD300a ITIM, but only SHP-1 is necessary for CD300a mediated inhibition

**Figure 5:**
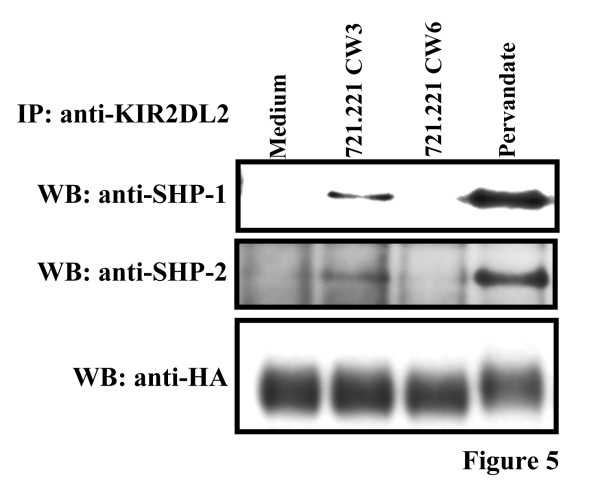
**The phosphatases SHP-1 and SHP-2 associate with tyrosine phosphorylated CD300a ITIMs.** KIR-CD300a Jurkat T cells were stimulated with medium or pervanadate for 3 minutes, or incubated for 5 minutes at 37°C with 721.221-Cw3 and 721.221-Cw6 cells. Then, cell lysates were immunopreciprecipitated with anti-KIR2DL2 (clone GL183) mAb and blotted separately for HA, SHP-1 and SHP-2. Results are representative of two independent experiments.

Tyrosine phosphorylation of ITIMs creates docking sites for SH2 domain containing proteins. ITIMs are known to specifically recruit phosphatases including SHP-1, SHP-2 and SHIP [13-16,40]. To identify potential phosphatases that bind to CD300a ITIMs, KIR-CD300a WT Jurkat T cells were treated with pervanadate or mixed with 721.221-Cw3 and 721.221-Cw6 cells and anti-KIR immunoprecipitates were probed with antibodies to SHP-1 and SHP-2. Both SHP-1 and SHP-2 coprecipitated with KIR-CD300a WT when cells were either treated with pervanadate or cocultured with 721.221-Cw3 cells but not 721.221-Cw6 cells (Figure 5). As expected, neither phosphatase coprecipitated with KIR-CD300a 4F (data not shown). Binding of SHIP to CD300a ITIMs could not be assessed in this system since Jurkat T cells do not express this phosphatase (data not shown).

**Figure 6:**
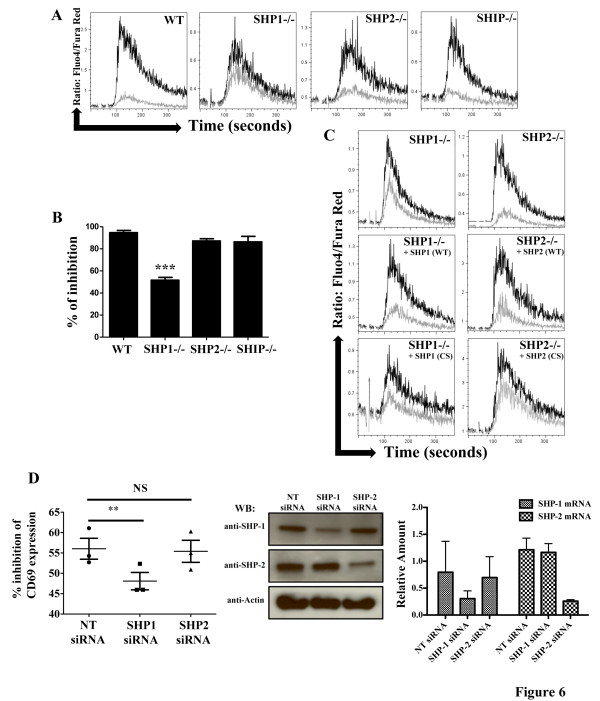
**SHP-1, but not SHP-2 or SHIP, is required for CD300a mediated inhibition of BCR stimulated activation.** (**A**) DT40 cells, DT40 cells lacking SHP-1, DT40 cells lacking SHP-2, or DT40 cells lacking SHIP, all expressing CD300a WT were loaded with Fluo-4 and Fura-Red. Then, cells were acquired in a flow cytometer and stimulated with anti-chicken BCR plus isotype control antibody (black line) or anti-chicken BCR plus anti-CD300a (grey line) mAb for 30 seconds and then co-crosslinked with a secondary antibody (GAM). Ca^2+^ mobilization is expressed as the ratio of Fluo-4/Fura-Red as a function of time. These results are representative of two independent experiments. (**B**) DT40 cell lines expressing CD300a WT were transiently transfected with a NFAT luciferase reporter plasmid and stimulated with anti-chicken BCR plus isotype control or anti-chicken BCR plus anti-CD300a mAb. Cells were lysed and supernatants assayed for luciferase activity. Results were normalized to the activity obtained when cells were treated with PMA plus ionomycin. Data are presented as percentage of inhibition of CD300a vs. isotype control and they are the average ± SEM for three separate experiments. (**C**) The indicated DT40 cells lines stably expressing CD300a WT and human SHP-1 WT, SHP-1 CS, SHP-2 WT or SHP-2 CS were loaded with Fluo-4 and Fura-Red. Ca^2+^ mobilization was assessed as in A. These results are representative of three independent experiments. (D) KIR-CD300a WT Jurkat T cells transfected with non target (NT), SHP-1 and SHP-2 siRNA were co-cultured with 721.221 or 721.221-Cw3 cells, loaded or not with SED. Cultures were harvested and Jurkat T cells were assessed for CD69 expression by flow cytometry. In the left panel, the percentage of inhibition of CD69 expression, calculated as shown in material and methods, is presented. The scatter plot represents the average ± SEM. In the center panel, the expression of SHP-1, SHP-2 and actin (loading control) was assessed by western blot analysis in lysates from siRNA transfected KIR-CD300a WT Jurkat T cells. In the right panel, the relative amount of SHP-1 mRNA and SHP-2 mRNA from siRNA transfected KIR-CD300a WT Jurkat T cells is shown. The bar graph represents the average ± SEM. Results are from three independent experiments.

In order to ascertain which of the phosphatases were responsible for the CD300a mediated inhibitory response, we made again use of the DT40 chicken B cells due to the availability of cell lines lacking SHP-1, SHP-2 or SHIP. Stable transfectants expressing CD300a WT were established in each of these cell lines and they were tested for inhibition of BCR stimulated Ca^2+^ mobilization. In SHP-2 and SHIP lacking DT40 chicken B cells, the coligation of the BCR with CD300a WT resulted in a decrease in the BCR stimulated rise of intracellular Ca^2+^ concentration similar to that obtained with wild type DT40 chicken B cells, suggesting that SHP-2 and SHIP do not have a primary role in the transmission of the CD300a inhibitory signal. On the other hand, the CD300a mediated inhibition of BCR induced Ca^2+^ mobilization was largely abolished in cells lacking SHP-1 (Figure 6A). The dominant role of SHP-1 in CD300a inhibitory signal was confirmed by a significant decrease in the CD300a mediated inhibition of BCR induced NFAT transcriptional activity in the SHP-1 deficient cells (Figure 6B). To further demonstrate the specific employment of SHP-1, we reconstituted SHP-1 deficient DT40 chicken B cells with human SHP-1 WT and SHP-1 CS. While the expression of human SHP-1 WT restored the inhibitory activity of CD300a, expression of SHP-1 CS, an inactive version of the phosphatase, did not (Figure 6C). We also reconstituted the SHP-2 deficient DT40 chicken B cells with human SHP-2 WT and SHP-2 CS. The expression of both human SHP-2 WT and SHP-2 CS resulted in a decrease in the CD300a mediated inhibition of BCR induced Ca^2+^ release when compared to SHP-2 deficient cells (Figure 6C). Finally, we efficiently suppressed the expression of SHP-1 and SHP-2 in the KIR-CD300a WT Jurkat T cells with specific siRNA. Results showed that while knock down of SHP-2 in KIR-CD300a WT Jurkat T cells has no effect in inhibiting CD69 induced expression after stimulation with 721.221-Cw3 cells loaded with SED, the SHP-1 knock down resulted in a decrease in the inhibitory potential of KIR-CD300a WT in suppressing CD69 induced expression after stimulation with SED loaded 721.221-Cw3 cells (Figure 6D). Taken together, these results indicate that although both SHP-1 and SHP-2 bind CD300a ITIMs, SHP-1 is the dominant phosphatase in the CD300a mediated signaling pathway, while SHP-2 and SHIP do not have a significant role.

## Discussion

In this report, we provide evidence that the primary function of CD300a in T and B cells is to limit antigen receptor mediated positive signaling and that the phosphatase SHP-1 is required for this function. Coligation of the BCR and CD300a with mAb reduced BCR stimulated Ca^2+^ mobilization and NFAT transcriptional activity. In the absence of SHP-1, but not SHP-2 or SHIP, CD300a mediated inhibition was significantly reduced. Additionally, we show that superantigen induced activation was inhibited when Jurkat T cells expressing the chimeric receptor KIR-CD300a were mixed with antigen presenting cells expressing the KIR2DL2 ligand HLA-Cw3. The interaction of KIR-CD300a with its ligand led to the tyrosine phosphorylation of CD300a ITIM motifs. This phosphorylation required the src kinase Lck, and provided docking sites for the binding of the phosphatases SHP-1 and SHP-2. These early events were followed by the inhibition of superantigen mediated up-regulation of activation markers CD25 and CD69.

The employment of two different models in attempting to understand CD300a inhibitory signal in lymphocytes was very important in our studies. Since DT40 chicken B cells do not express CD300a, the usage of these cells and their knockout counterparts allowed us to specifically express both CD300a and phosphatases, wild type and mutants, and to investigate the role of phosphatases in CD300a signaling transmission. In our hands, and with the available anti-CD300a mAb, we were unable to immunoprecipitate CD300a (data not shown). Due to this inability to immunoprecipitate CD300a and, because we also were interested in an experimental system that relies on receptor-ligand interaction, we generated the chimeric receptor KIR-CD300a. Results obtained with similar chimeric receptors have proved useful in gaining information about the role of the ITIMs. For example, by using a chimeric receptor consisting of KIR extracellular domain fused to the FcγRIIb intracellular tail, Gupta et al. demonstrated that the ITIMs in the intracellular tail, and not the extracellular portion, are responsible for the transmission of the inhibitory signal and determined which phosphatase was employed [41].

For a more comprehensive understanding of CD300a mediated signaling on lymphocytes, mutational analysis of the ITIMs should prove helpful. Lankry et al. have undertaken these studies using the human YTS NK cell line [29]. Their results indicated that all of the ITIMs, including the non-classical 4^th^ ITIM, were important for the inhibitory function of CD300a, with the 3^rd^ ITIM being the most essential. Results obtained in our laboratory in which we mutated tyrosine residues to phenlyalanine instead of to alanine, as described by Lankry et al. [29], have confirmed that a single mutation of the 3^rd^ ITIM significantly decreased BCR stimulated Ca^2+^ release and NFAT transcriptional activity (data not shown).

In our KIR-CD300a chimera, the CD300a ITIMs were phosphorylated upon interaction with the KIR ligand without the requirement of superantigen stimulation. This is not surprising, since phosphorylation of KIR ITIMs by Lck also occurs independently of antigen stimulation [9,42]. However, it is interesting that a single tyrosine kinase, such as Lck, can be utilized for both inhibitory and activating receptors. While the mechanism by which this occurs is still under investigation, findings obtained by Stefanova et al. [43] may shed some light on this conundrum. In that report, antagonist and agonist peptides, defined by their different binding affinities to the TCR, were used to dissect the seemingly different roles of Lck in T cell homeostasis. The SHP-1 tyrosine phosphatase was a central player in their findings. When T cells were stimulated with a weak binding ligand, Lck phosphorylated SHP-1. Subsequent association of SHP-1 with Lck mediated the recruitment of SHP-1 to the TCR complex where it was proposed that SHP-1 then dephosphorylated Lck at Y394 leading to TCR desensitization. Alternatively, upon interaction with a strong TCR ligand, Erk was rapidly activated and phosphorylated Lck on serine residues (S59). This serine phosphorylation decreased the ability of Lck to bind SHP-1 and therefore the positive signaling proceeded. Here, we have shown that Lck is involved in CD300a phosphorylation. It may be possible that Lck also phosphorylates CD300a bound SHP-1, subsequently aiding in the recruitment of SHP-1 to the TCR complex, leading to the inhibition of positive signaling. Future studies should address this hypothesis.

Our results using SHP-1 and SHP-2 knocked down KIR-CD300a WT Jurkat T cells and specific phosphatase deficient DT40 chicken B cells indicated that SHP-1, but not SHP-2 or SHIP was necessary for CD300a mediated inhibition of BCR and TCR signaling. Although mAb cross-linking induced coimmunoprecipitation of SHIP with CD300a in mast cells [30], the consensus binding sequences for SHIP are different from that of SHP-1 and SHP-2 and are not present in CD300a. SHIP has no preference for binding to residues N-terminal to the phosphorylated tyrosine (pY) but has a strong preference for Leu at the +2 position. Instead, SHP SH2 domains prefer a hydrophobic residue at the −2 position on the ITIM [44]. All three classical ITIMs present in CD300a have hydrophobic residues at −2 and none of them have Leu at +2 position [28]. Therefore, while the detection of SHIP in a complex with CD300a may indicate a role for SHIP in the control of signaling in mast cells, its direct binding to CD300a ITIM motifs is unlikely. On the other hand, the consensus binding motifs for SHP-1 and SHP-2 are similar [44] and matched sequences are found in the CD300a intracellular tail [28]. Indeed, both SHP-1 and SHP-2 were detected in immunoprecipitates from ligand stimulated Jurkat T cells expressing the KIR-CD300a chimeric receptor. However, according to previously published results that tested the binding of SHP SH2 domains to pY peptide libraries [44], it may be that the chances of having both SH2 domains of a single phosphatase bound simultaneously to phosphorylated CD300a intracellular tail are greater with SHP-1. While binding of a single SH2 domain may potentiate phosphatase activity, binding of both domains further increases the activity by several fold [45].

Further evidence that both SHP-1 and SHP-2 bind to CD300a comes from the SHP-1 and SHP-2 reconstitution experiments. As shown in Figure 6, the expression of either SHP-1 CS or SHP-2 CS reduced the inhibitory function of CD300a. The mutation of the cysteine residue renders the phosphatases catalytically inactive, but they still are able to bind the target ITIM sequences and therefore become dominant negative. In that same line of thought, one could argue that since SHP-2 WT also competes for CD300a ITIM occupancy, it could also function as a dominant negative, and in fact, reconstitution of CD300a expressing DT40 chicken B cells lacking SHP-2 with human SHP-2 WT resulted in a decrease in the CD300a mediated inhibitory ability when compared with non-reconstituted DT40 cells lacking SHP-2 (see Figure 6C). Additional studies designed to address the relative binding affinity of SHP-1 and SHP-2 to phosphorylated CD300a ITIMs and their differential role in signaling should prove interesting.

## Conclusions

Taken together, we have demonstrated that CD300a inhibits lymphocyte immune receptor signaling *via* SHP-1. Although both SHP-1 and SHP-2 are recruited to the phosphorylated ITIMs of CD300a, only the absence of SHP-1 limited the ability of CD300a to inhibit activation signals. SHP-1 has historically been associated with negative signaling while SHP-2 has been associated with positive signals [18]. Beyond similarities in their SH2 domains, the two phosphatases display little sequence homology. SHP-1 but not SHP-2 displays localization signals in particular for lipid rafts while only SHP-2 has proline-rich domains which could recruit SH3 domain containing proteins [44]. However, the specific roles of these regions may play in the function of the two phosphatases remains to be defined. With this in mind, the availability of the different phosphatases to bind the CD300a intracellular tail following receptor ligation will determine the final outcome of the CD300a mediated signaling.

## Methods

### Cells and reagents

The E6.1 Jurkat T cell line, the Jurkat T cell lines deficient in Lck (JCaM1.6) [46] and ZAP-70 (P116) [47], the DT40 chicken B cell line [48], the DT40 mutant cell lines lacking SHP-1, SHP-2 and SHIP [14,49], and the MHC class I deficient human lymphoblastoid B cell line 721.221 and its clones expressing HLA-Cw3 and -Cw6 [50,51] were all maintained in RPMI medium containing 7.5% FBS.

To generate stable transfectants, 1 x 10^7^ DT40 chicken B cells or E6.1 Jurkat T cells were transfected with the designated plasmids by electroporation. For DT40 chicken B cells, in addition to the CD300a and phosphatase expressing plasmids, cells were also transfected with 5 μg of the pBABE puro vector [52]. After 48 hours in complete medium, cells were selected with neomycin (Invitrogen) or puromycin (Sigma). Cells were tested for CD300a expression, sorted using a FACS Aria sorter (BD Biosciences) and positive cells were further expanded. Cells transfected with plasmids expressing both CD300a and phosphatases were preselected for CD300a expression, then cloned and tested by Western blot for phosphatase expression. All transfected DT40 chicken B cells and Jurkat T cells expressed similar levels of CD300a and KIR-CD300a, respectively (data not shown).

Antibodies used in this study were obtained from the following vendors: PE-Cy7 anti-CD19 (clone HIB19), Alexa Fluor 488 anti-CD25 (clone BC96), PE anti-CD69 (clone FN50) and isotype control murine IgG1κ were purchased from eBioscience; purified anti-CD158b (clone GL183), PE and purified anti-CD300a (clone E59.126) were purchased from Beckman Coulter; PE anti-CD158b (clone DX27) was purchased from BioLegend; purified anti-chicken IgM (clone M1) was purchased from Southern Biotech; FITC goat anti-mouse IgG F(ab’)_2_ was purchased from KPL; anti-HA high affinity (clone 3F10) and 3F10-HRP were purchased from Roche-Diagnostics; rabbit anti-mouse IgG was purchased from MP Biomedicals; goat anti-mouse IgG was purchased from Jackson ImmunoResearch Laboratories; anti-phosphotyrosine (clone 4G10), biotinylated 4G10, and anti-SHP-1 antibodies were from Millipore; anti-SHP-2 (clone 79/PTP1D/SHP2) was from BD Biosciences; anti-mouse HRP and anti-rabbit HRP were purchased from GE Healthcare and NeutrAvidin-HRP was from Fisher. SHP-1 specific siRNA duplexes were from Sigma, and non-target (NT) and SHP-2 specific siRNA duplexes were from Dharmacon.

A plasmid encoding human CD300a was previously described [25]. The KIR-CD300a chimeric construct was engineered by fusing the extracellular domain of the KIR2DL2 receptor to the transmembrane and cytoplasmic domains of CD300a tagged with HA (Figure 2). A KIR2DL2 expressing plasmid (a generous gift of Dr. Eric O. Long) was used as a template for the extracellular domain of KIR2DL2. The following primers were used for the PCR reaction: a forward primer 5’ GGGGTACCGCCGCCATGTCGCTCATGGTCG 3’ and a reverse primer 5’ GAAGATCTGTGCAGGTGTCGGGG 3’. A pMACS CD300a-HA plasmid (engineered in our laboratory) was used as a template for the transmembrane and cytoplasmic tail of CD300a tagged with HA. The following primers were used for the PCR reaction: a forward primer 5’ GAAGATCTCTCTGCTCCTCTCCCTGC 3’ and a reverse primer 5’ GCTCTAGATCATTAAGCGTAGTCTGG 3’. The PCR products were purified and digested with the restriction enzymes Kpn I/Bgl II and Bgl II/Xba I, respectively. Digested products were ligated into an empty pcDNA3.1 mammalian expression vector digested with Kpn I and Xba I. Mutagenesis of CD300a and KIR-CD300a expressing plasmids were performed with specific primers using a Quick Change Site-directed mutagenesis kit (Stratagene). DNA sequencing analysis confirmed the sequences of each construct. Plasmids expressing human SHP-1 and SHP-2, wild type (WT) and the catalytically inactive (CS mutation), were a generous gift of Dr. Eric O. Long.

### Cell activation, immunoprecipitation and western blot analyses

Jurkat T cells, 5–10 x 10^6^, were prewarmed at 37°C, treated with 0.1 mM sodium orthovanadate and 0.3 mM hydrogen peroxide for 3–5 minutes and then lysed with 50 mM Tris HCl containing 1% NP40 plus protease and phosphatase inhibitors as previously described [53]. In other experiments, Jurkat T cells were mixed with an equal number of the indicated antigen presenting cells (721.221 cells), in the absence or presence of staphylococcal enterotoxin D (SED) (Toxin Technology) at 100 ng/ml while maintained on ice and then centrifuged to promote cell to cell contact. The supernatants were removed and the cell pellets were incubated at 37°C and then, cells were lysed as described above. For immunoprecipitation experiments, cell lysates were precleared with Protein A/G beads (Pierce) for one hour followed by precipitation with Protein A/G beads preloaded with 1.6 μg of anti-KIR2DL2 (clone GL183). Eluted proteins were resolved on gradient gels (Invitrogen), transferred to nitrocellulose and probed with the indicated antibodies.

### Flow cytometry experiments

Jurkat T cells, 1 x 10^6^, were mixed with an equal number of 721.221 cells and distributed in wells of a 12 well plate without or with 100 ng/ml SED. After 24 h of culture, the expression of CD25 and CD69 by the Jurkat T cells was assessed by flow cytometry using a FACS Calibur (BD Biosciences). Jurkat T cells were electronically gated by size and forward scatter and by their lack of expression of CD19, a marker expressed by the lymphoblastoid human B cell line 721.221. In the knock down experiments, prior to the mixing with 721.221 and 721.221-Cw3 cells, KIR-CD300a WT Jurkat T cells were transfected with 400 μM of the indicated siRNA duplexes using the Amaxa Nucleofection System (Lonza). After 36 hours, the efficiency of the knock down was measured at the mRNA and protein levels by real-time PCR and western blot analysis, respectively. The percentage of inhibition of CD69 expression was calculated according to the following formula: [(SED loaded 721.221 Mediated CD69 induction – SED loaded 721.221-Cw3 Mediated CD69 Induction)/SED loaded 721.221 Mediated CD69 induction] x 100. CD69 expression was measured by median fluorescence intensity (MFI).

### NFAT luciferase reporter assays

DT40 chicken B cells expressing CD300a or Jurkat T cells expressing the chimeric KIR-CD300a receptor were transiently transfected with 5 μg of an NFAT luciferase reporter construct and cultured for 16 hours. For experiments with B cells, DT40 transfectants were distributed into duplicate wells of a 24 well plate containing medium alone, prebound anti-mouse IgG plus anti-chicken IgM and either isotype IgG1 control antibody or anti-CD300a mAb. For experiments with T cells, Jurkat transfectants were mixed with an equal number of 721.221 cells and added to wells of a 24 well plate with or without the superantigen, SED (100 ng/ml). As a measure for maximal NFAT activity, cells were treated with 50 ng/ml phorbol myristate acetate (PMA) plus 5 μM ionomycin, purchased from EMD. After 6 h, cells were disrupted in lysis buffer (Promega) and lysates were assayed using luciferin (Promega).

### Calcium mobilization assays

DT40 chicken B cells, 1.5 x 10^6^, were washed with PBS containing 1% BSA, resuspended in 3 ml and loaded with 3 μg of Fluo-4 and 7.5 μg of Fura-Red (Invitrogen) for 30 minutes at 30°C. Then, cells were washed twice and aliquots of 1 ml were warmed at 37°C for 5 minutes, followed by acquisition in a flow cytometer (FACS Calibur). To establish a baseline, cells were first acquired for 30 seconds, at which point the anti-IgM mAb plus the anti-CD300a mAb or isotype control IgG1 were added and acquisition was followed for another 30 seconds. Then, the secondary antibody was added and acquisition was followed for 5–6 minutes. Data were analyzed using the FlowJo software (Treestar).

### Statistical analysis

Data were analyzed using GraphPad Prism software. The data were plotted as bar graphs or scatter plots, and pair wise comparisons were examined by two-tailed paired Student’s *t*-test. NS: non significant; * P < 0.05, ** P < 0.01; *** P < 0.001.

## Competing interests

The authors declare that they have no competing interests.

## Authors’ contributions

KED and FB designed the study. KED, VRS, FB, and JLM performed the experiments, collected and analyzed data. KED and FB wrote the manuscript. All authors read and approved the final manuscript.
